# Synergistic use of glycomics and single‐molecule molecular inversion probes for identification of congenital disorders of glycosylation type‐1

**DOI:** 10.1002/jimd.12496

**Published:** 2022-03-28

**Authors:** Nurulamin Abu Bakar, Angel Ashikov, Jaime Moritz Brum, Roel Smeets, Marjan Kersten, Karin Huijben, Wee Teik Keng, Carlos Eduardo Speck‐Martins, Daniel Rocha de Carvalho, Isabela Maria Pinto Oliveira de Rizzo, Walquiria Domingues de Mello, Rebecca Heiner‐Fokkema, Kathleen Gorman, Stephanie Grunewald, Helen Michelakakis, Marina Moraitou, Diego Martinelli, Monique van Scherpenzeel, Mirian Janssen, Lonneke de Boer, Lambertus P. van den Heuvel, Christian Thiel, Dirk J. Lefeber

**Affiliations:** ^1^ Department of Neurology, Translational Metabolic Laboratory, Donders Institute for Brain, Cognition, and Behavior Radboud University Medical Center Nijmegen The Netherlands; ^2^ Department of Pathology Selayang Hospital, Selangor, Ministry of Health Malaysia Putrajaya Malaysia; ^3^ Department of Clinical Pathology The Sarah Network of Rehabilitation Hospitals Brasilia Brazil; ^4^ Translational Metabolic Laboratory, Department Laboratory Medicine Radboud University Medical Center Nijmegen The Netherlands; ^5^ Genetics Department Kuala Lumpur Hospital, Kuala Lumpur, Ministry of Health Malaysia Putrajaya Malaysia; ^6^ Genetic Unit Sarah Network of Hospitals Brasilia Brazil; ^7^ Department of Laboratory Medicine UMC Groningen Groningen The Netherlands; ^8^ Pediatric Neurology Children's Health Ireland (CHI) Dublin Ireland; ^9^ Metabolic Department, Great Ormond Street Hospital NHS Foundation Trust Institute of Child Health University College London London UK; ^10^ Department of Enzymology and Cellular Function Institute of Child Health Athens Greece; ^11^ Genetics and Rare Diseases Research Division Bambino Gesù Children's Research Hospital Rome Italy; ^12^ Department of Internal Medicine Radboud University Medical Center Nijmegen The Netherlands; ^13^ Department of Pediatrics Radboud University Medical Center Nijmegen The Netherlands; ^14^ Department of Pediatrics, Translational Metabolic Laboratory Radboud University Medical Center Nijmegen The Netherlands; ^15^ Center for Child and Adolescent Medicine Kinderheilkunde I, University of Heidelberg Heidelberg Germany

**Keywords:** CDG type 1 (CDG‐I), congenital disorders of glycosylation (CDG), diagnostics by mass spectrometry, glycomics, multi‐omics, smMIPs

## Abstract

Congenital disorders of glycosylation type 1 (CDG‐I) comprise a group of 27 genetic defects with heterogeneous multisystem phenotype, mostly presenting with nonspecific neurological symptoms. The biochemical hallmark of CDG‐I is a partial absence of complete N‐glycans on transferrin. However, recent findings of a diagnostic N‐tetrasaccharide for ALG1‐CDG and increased high‐mannose N‐glycans for a few other CDG suggested the potential of glycan structural analysis for CDG‐I gene discovery. We analyzed the relative abundance of total plasma N‐glycans by high resolution quadrupole time‐of‐flight mass spectrometry in a large cohort of 111 CDG‐I patients with known (*n* = 75) or unsolved (*n* = 36) genetic cause. We designed single‐molecule molecular inversion probes (smMIPs) for sequencing of CDG‐I candidate genes on the basis of specific N‐glycan signatures. Glycomics profiling in patients with known defects revealed novel features such as the N‐tetrasaccharide in ALG2‐CDG patients and a novel fucosylated *N*‐pentasaccharide as specific glycomarker for ALG1‐CDG. Moreover, group‐specific high‐mannose N‐glycan signatures were found in ALG3‐, ALG9‐, ALG11‐, ALG12‐, RFT1‐, SRD5A3‐, DOLK‐, DPM1‐, DPM3‐, MPDU1‐, ALG13‐CDG, and hereditary fructose intolerance. Further differential analysis revealed high‐mannose profiles, characteristic for ALG12‐ and ALG9‐CDG. Prediction of candidate genes by glycomics profiling in 36 patients with thus far unsolved CDG‐I and subsequent smMIPs sequencing led to a yield of solved cases of 78% (28/36). Combined plasma glycomics profiling and targeted smMIPs sequencing of candidate genes is a powerful approach to identify causative mutations in CDG‐I patient cohorts.

## 1 INTRODUCTION

Congenital disorders of glycosylation (CDG) form a large group of inherited diseases with extremely broad spectrum of clinical symptoms. Since its first description in 1980, more than 140 types of CDG have been reported of which 70 types with deficient *N*‐linked protein glycosylation.^1^ CDG type 1 (CDG‐I) are seen as the classical form of CDG and comprise defects in the endoplasmic reticulum N‐glycosylation pathway. CDG‐I patients generally present with multisystem clinical phenotypes with the majority affected by neurological symptoms. Clinical clues might be useful to diagnose CDG‐I defects such as (a) nonneurological involvements in MPI‐CDG (gastrointestinal/liver phenotype) and DPM3‐CDG (heart and muscle phenotype); (b) ichthyosis in MPDU1‐CDG, DOLK‐CDG, and SRD5A3‐CDG; (c) neurosyndromic cataract and/or coloboma in SRD5A3‐CDG and ALG2‐CDG; and (d) neurosyndromic sensorineural deafness in ALG11‐CDG and RFT1‐CDG.^2^ Traditionally, plasma transferrin has been used as the diagnostic protein marker to screen for CDGs with deficient N‐glycosylation. Defects in CDG‐I result in a partial absence of complete glycans on the transferrin protein. Introduction of plasma intact transferrin mass spectrometry (MS) has significantly improved the identification of CDG‐I patients due to the sensitive detection of a glycan loss.^3^, ^4^ To date, at least 27 different genetic defects are known that result in CDG‐I screening profiles.^5^ Further confirmation of CDG‐I gene defects has long depended on enzymatic assays in blood cells, analysis of dolichol‐linked oligosaccharide (DLO) in patient fibroblasts or additional biochemical tests, such as analysis of dolichol metabolites in plasma or urine. In recent years, this has largely been replaced by genomics techniques, such as whole exome sequencing (WES) or targeted panel sequencing of CDG‐I genes.^6^ Still, fast and specific biochemical tests are warranted to guide CDG‐I gene identification for gene‐specific sequencing, like in countries or circumstances where WES is less accessible, or to provide functional validation of mutations in candidate genes. In recent years, an abnormal glycan structure, coined N‐tetrasaccharide, was identified in plasma as diagnostic marker for ALG1‐CDG and was also observed in PMM2‐CDG and MPI‐CDG patients.^7^ Subsequent studies revealed the robustness of this marker in a cohort of 39 previously unreported cases of ALG1‐CDG patients.^8^ Recently, a novel semiquantitative N‐glycan assay by electrospray ionization quadrupole time‐of‐flight (QTOF) MS was developed to improve the clinical specificity and sensitivity of N‐glycan profiling.^9^ This indicated an opportunity to position plasma N‐glycan profiling in CDG‐I diagnostics. In synergy with modern genomics technologies, specific glycomics signatures can help to confirm a genetic diagnosis and to unravel novel gene defects, as recently proven in a cohort of CDG‐II patients with abnormal Golgi N‐glycosylation defects (CDG‐II).^10^ In addition to the targeted sequencing of gene panels by WES or multiple polymerase chain reactions (PCRs) and Sanger/Next Generation Sequencing, introduction of single‐molecule molecular inversion probes (smMIPs) offered a fast, robust, and cheap approach for targeted sequencing of candidate genes.^11^

Here, we performed plasma glycomics in a comprehensive cohort of 111 of CDG‐I patients. Abnormal glycan structures were found for 16 of the 20 gene defects that were involved in this study. Application of glycomics in a cohort of 36 unsolved CDG‐I patients revealed a characteristic glycan profile, which allowed for selection of genes for targeted smMIPs sequencing and identification of the genetic cause in 28 patients.

## 2 MATERIALS AND METHODS

### 2.1 Samples and subjects

Plasma samples of patients with a CDG‐I screening profile due to a known (*n* = 71), secondary (*n* = 4), or unsolved genetic cause (*n* = 36; unsolved at the time of enrolling in the glycomics study) (see Table S1) were obtained from the diagnostic archives of the (a) Radboud UMC, Nijmegen, The Netherlands, (b) University of Heidelberg, Germany, and (c) UMC Groningen, The Netherlands, and used in accordance with Helsinki's Declaration.

### 2.2 Glycosylation analyses

Plasma of patients was analyzed for transferrin N‐glycosylation by isofocusing and C8‐chip‐QTOF MS as described.^12^ For the analysis of N‐glycans from total plasma proteins, 10 μl of thermally denatured plasma sample was treated with peptide *N*‐glycosidase F (PNGaseF), followed by the extraction and analysis of N‐glycans using a porous graphitized carbon chip and an Agilent 6540 QTOF mass spectrometer, as described.^10^, ^13^, ^14^ Data analysis for total plasma N‐glycan profiling was performed using Agilent Mass Hunter Qualitative Analysis Software B.05 using the Molecular Feature Extractor with signal‐to‐noise ratio of 5. Only human N‐glycan compositions were included consisting of (a) hexose (Hex); for example, mannose (Man), galactose (Gal), and glucose (Glc); (b) *N*‐acetylhexosamine (HexNAc); for example, *N*‐acetylglucosamine (GlcNAc); (c) deoxyhexose (dHex); for example, fucose (Fuc); and (d) *N*‐acetylneuraminic acid/sialic acid (Neu5Ac/Sia). Relative abundances of each glycan were obtained from a single run analysis through normalization to the total signal of all detected N‐glycans. Data were analyzed using GraphPad Prism (version 5.03) and IBM SPSS (version 22.0) software. A parametric approach using central 95% confidence intervals (CIs) of the control group (*n* = 40) was used to express the reference limits of total plasma N‐glycans, as described.^10^ Student's *t*‐test was performed to evaluate statistical significance of glycans' relative abundances between two groups. The *p* values of ≤0.05 were considered significant.

### 2.3 Genomics study

#### 2.3.1 Whole exome sequencing

High molecular weight genomic DNA was isolated using Blood & Cell culture DNA Mini Kit (Qiagen) following the manufacturer protocol. Barcoded libraries were prepared by the SureSelect Human All Exon 50 Mb v4 kit (Agilent Technologies) and then sequenced on a SOLiD 5500xl sequencer (Life Technologies) with 50‐bp single fragment read. Raw reads were aligned onto the GRCh37 (hg19) reference genome with the SOLiDLifeScope software version 2.1. Variants and indels annotations were completed using an in‐house pipeline. Routine screening for recessive monogenic disease based on selection of variants with a frequency below 0.05% was performed. Synonymous variants, deep intronic variants, and variants in untranslated regions were excluded from the list of potentially causative genes.

#### 2.3.2 Sanger sequencing for ALG1‐CDG

To circumvent homologues, all coding exons including the intron/exon boundaries of the *ALG1* gene (NCBI Reference Sequence NM_019109) were analyzed by PCR and Sanger Sequencing. High molecular weight genomic DNA was isolated using Blood & Cell culture DNA Mini Kit (Qiagen) following the manufacturer protocol. PCR was performed using the AmpliTaq Gold™ 360 Master Mix (ThermoFisher Scientific; primer sequences and thermal cycling conditions available upon request), and PCR products were purified by a MultiScreen‐PCR96 Filter Plate and MultiScreen™ Vacuum Manifold 96‐well (Merck Millipore) as described by the manufacturers. Sequencing analysis was performed using the BigDye™ Terminator v1.1 Cycle Sequencing Kit and a 3730xl DNA Analyzer (ThermoFisher Scientific) following the recommendations of the manufacturer. Sanger sequences were aligned onto the NCBI Reference Sequence and analyzed with the SeqPatient module of SeqPilot 4.4.0 (JSI Medical Systems).

#### 2.3.3 Single‐molecule molecular inversion probes

All coding exons including the intron/exon boundaries of the *ALG3* (NM_005787), *ALG6* (NM_013339), *ALG11* (NM_001004127), *ALG13* (NM_001099922/NM_018466), *DDOST* (NM_005216), *DOLK* (NM_014908), *DPAGT1* (NM_001382), *DPM1* (NM_003859), *DPM2* (NM_003863), *DPM3* (NM_018973), *MPDU1* (NM_004870), *MPI* (NM_002435), *PMM2* (NM_000303), *RFT1* (NM_052859), *SRD5A3* (NM_024592), *SSR4* (NM_001204526), *STT3A* (NM_001278503), and *STT3B* (NM_178862) genes were analyzed by smMIPs enrichment and NGS Sequencing on a NextSeq500 (Illumina). High molecular weight genomic DNA was isolated using Blood & Cell culture DNA Mini Kit (Qiagen) following the manufacturer protocol. The smMIP design and protocol was based on previous methods, and modifications of this protocol and its implementation to an automated work flow were developed in‐house (smMIP sequences available upon request).^15^, ^16^ Raw reads were aligned onto the matching NCBI Reference Sequence and analyzed with the SeqNext module of SeqPilot 4.4.0 (JSI Medical Systems).

## 3 RESULTS

### 3.1 Identification of N‐tetrasaccharide in ALG2‐CDG, and a novel *N*‐pentasaccharide glycan as marker for ALG1‐CDG

**FIGURE 1:**
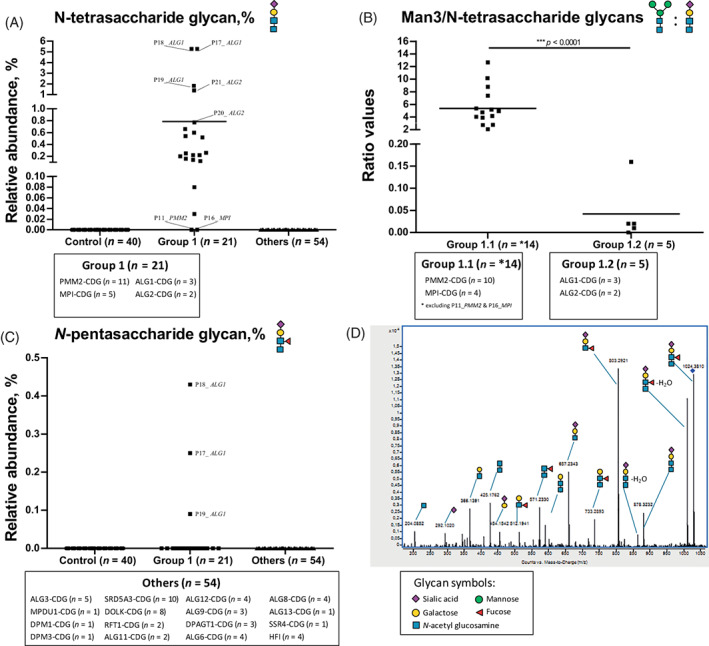
Total plasma glycoprofiling of N‐tetrasaccharide glycan for subtyping of Group 1 (PMM2‐, MPI‐, ALG1‐, and ALG2‐congenital disorders of glycosylation [CDG]) and *N*‐pentasaccharide glycan for direct diagnosis of ALG1‐CDG. (A) Relative abundance of the N‐tetrasaccharide in Control, Group 1, and other CDG type 1 (CDG‐I) defects (Others). High abundance of this glycomarker was proposed to screen for Group 1; (B) further differential analysis of Group 1 using the ratio of the Man3/N‐tetrasaccharide glycans distinguished between Group 1.1 (PMM2‐CDG and MPI‐CDG) and Group 1.2 (ALG1‐CDG and ALG2‐CDG); (C) relative abundance of the *N*‐pentasaccharide in Control, Group 1, and Others. This novel glycomarker is specific for direct diagnosis of ALG1‐CDG; and (D) fragmentation of the *N*‐pentasaccharide glycan by quadrupole time‐of‐flight (QTOF) MS/MS analysis

Plasma glycomics was performed in a comprehensive CDG‐I cohort, consisting of 75 patients covering 20 gene defects. Glycans were expressed as the relative abundance of total plasma N‐glycans (Table S1). As depicted in Figure 1A, we identified the N‐tetrasaccharide glycan (Neu5Ac_1_Gal_1_GlcNAc_2_) in ALG2‐CDG (*n* = 2) as well as in the previously described group of ALG1‐, PMM2‐, and MPI‐CDG patients.^7^ In view of the presence of the N‐tetrasaccharide glycan, we categorized the group of PMM2‐, MPI‐, ALG1‐, and ALG2‐CDG as *Group 1*. Simultaneously, we did not detect the N‐tetrasaccharide glycan in P11 (PMM2‐CDG) and P16 (MPI‐CDG) (Figure 1A) indicating that the presence of this glycan is not prerequisite for diagnosis of PMM2‐ and MPI‐CDG. We performed further differential analysis for *Group 1* (Figure 1B) using the ratio of Man3 (Man_3_GlcNAc_2_)/N‐tetrasaccharide glycans, as described.^7^ This allowed to distinguish the group of PMM2‐ and MPI‐CDG patients (*Group 1.1*) from patients with ALG1‐ and ALG2‐CDG (*Group 1.2*).

Interestingly, we identified a novel *N*‐pentasaccharide (Neu5Ac_1_Gal_1_(Fuc)GlcNAc_2_) (1024.38 m/z) in all patients with ALG1‐CDG (*n* = 3) and not in other patients with the N‐tetrasaccharide (Figure 1C) suggesting that this glycan could serve as a specific glycomarker for ALG1‐CDG. Fragmentation by MS/MS showed characteristic fragment ions for the presence of fucose linked to the nonterminal GlcNAc residue and not to the core GlcNAc residue. Thereby, the composition of this novel glycan was determined as a sialylated and fucosylated *N*‐pentasaccharide glycan (Figure 1D). This structure is in agreement with the proposed biosynthesis pathway, in which the chitobiose disaccharide (GlcNAc_2_) is attached to proteins in the endoplasmic reticulum (ER) and further extended by Golgi glycosyltransferases.^7^ Since the novel *N*‐pentasaccharide glycan was not detected in all patients with ALG2‐CDG (*n* = 2), we hypothesize that the ratio of Man3 glycan/N‐tetrasaccharide glycan could be implemented to discriminate ALG2‐CDG from other patients in *Group 1.1*. In this study, we could not identify the plasma *N*‐pentasaccharide glycan in *Control* (*n* = 40), in all patients with ALDOB deficiency/hereditary fructose intolerance (HFI) (*n* = 4), PMM2‐CDG (*n* = 11), MPI‐CDG (*n* = 5), ALG3‐CDG (*n* = 5), MPDU1‐CDG (*n* = 1), DPM1‐CDG (*n* = 1), DPM3‐CDG (*n* = 1), SRD5A3‐CDG (*n* = 10), DOLK‐CDG (*n* = 8), RFT1‐CDG (*n* = 2), ALG11‐CDG (*n* = 2), ALG12‐CDG (*n* = 4), ALG13‐CDG (*n* = 1), ALG9‐CDG (*n* = 3), ALG6‐CDG (*n* = 4), ALG8‐CDG (*n* = 4), DPAGT1‐CDG (*n* = 3), and SSR4‐CDG (*n* = 1) as well as in patients from our previous glycomics cohort of PGM1‐CDG and CDG type 2 (CDG‐II).^10^, ^13^

### 3.2 Identification of abnormal high‐mannose N‐glycan profiles for several CDG‐I types, including ALG9‐ and ALG12‐CDG

**FIGURE 2:**
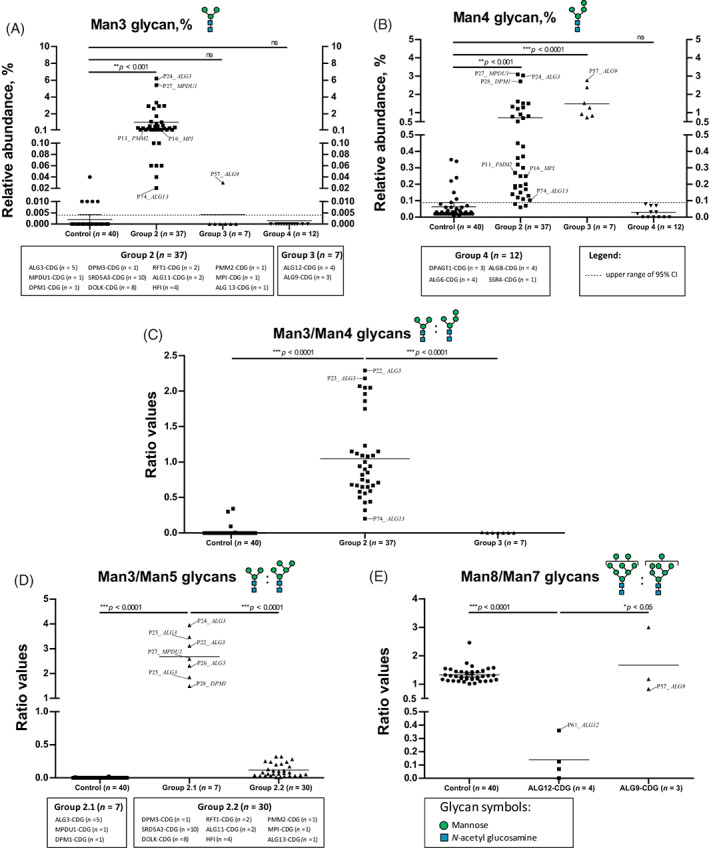
Total plasma glycoprofiling of several high‐mannose N‐glycans for subtyping of Group 2 (ALG3‐, MPDU1‐, DPM1‐, DPM3‐, SRD5A3‐, DOLK‐, RFT1‐, ALG11‐, ALG13‐, PMM2‐, MPI‐congenital disorders of glycosylation [CDG], and hereditary fructose intolerance [HFI]), Group 3 (ALG12‐ and ALG9‐CDG), and Group 4 (CDG‐I defects other than Groups 1–3). High relative abundance of Man3 glycan (A) and Man4 glycan (B) in CDG‐I with mannosylation defects including Groups 2 and 3, as compared with Control and Group 4; (C) further ratio analysis of relative abundances of Man3/Man4 glycans allow discrimination of Groups 2 and 3; (D) analysis of the Man3/Man5 ratio could be used to differentiate Group 2.1 (ALG3‐, MPDU1‐, and DPM1‐CDG) and Group 2.2 (DPM3‐, SRD5A3‐, DOLK, RFT1‐, ALG11‐, ALG13‐, PMM2‐, MPI‐CDG, and HFI); and (E) the relative abundance of Man7/Man8 glycans can be used as diagnostic glycoprofile for ALG12‐ and ALG9‐CDG (Group 3)

Next, we focused on a group of patients with high abundance of high‐mannose Man3 (Figure 2A, Group 2) and Man4 glycans (Man_4_GlcNAc_2_) (Figure 2B, Group 3). As seen in Figure 2C, the ratio of Man3/Man4 glycans allowed discrimination between Groups 2 and 3. *Group 2* includes patients with ALG3‐CDG (*n* = 5), MPDU1‐CDG (*n* = 1), DPM1‐CDG (*n* = 1), DPM3‐CDG (*n* = 1), SRD5A3‐CDG (*n* = 10), DOLK‐CDG (*n* = 8), RFT1‐CDG (*n* = 2), ALG11‐CDG (*n* = 2), ALG13‐CDG (*n* = 1), HFI (*n* = 4), and also PMM2‐CDG (P11) and MPI‐CDG (P16). *Group 3* included ALG12‐CDG (*n* = 4) and ALG9‐CDG (*n* = 3), which showed an extremely low ratio (range: 0–0.005). In *Group 3*, we found that the Man3 glycan was completely absent in the majority of patients (except P57_*ALG9*) while the Man4 glycan was high in abundance as compared to controls.

To further subdivide patients within *Group 2*, we calculated the ratio of Man3/Man5 (Man_5_GlcNAc_2_) glycans, the Man5 glycan being the most abundant high‐mannose glycan in controls. As seen in Figure 2D, this ratio allowed discrimination of a group of patients with ALG3‐, MPDU1‐, and DPM1‐CDG (*Group 2.1*) from a group of patients with DPM3‐, SRD5A3‐, DOLK, RFT1‐, ALG11‐, ALG13‐, PMM2‐, MPI‐CDG, and HFI (*Group 2.2*). Finally, ALG12‐ and ALG9‐CDG could be discriminated by taking the ratio of Man8 (Man_8_GlcNAc_2_)/Man7 (Man_7_GlcNAc_2_) glycans. This is in agreement with the known accumulation of these high‐mannose glycans in DLO analysis in patient fibroblasts. As depicted in Figure 2E, the differential analysis of *Group 3* by calculating the Man8/Man7 ratio could successfully discriminate both gene defects. All ALG12‐CDG patients (*n* = 4) exhibited low values (range: 0–0.4) as compared to control (range: 1.0–2.5) and ALG9‐CDG (range: 0.8–3.0). Finally, *Group 4* included patients with normal glycan profiles, including the *N*‐tetrasaccharide, *N*‐pentasaccharide, and high‐mannose glycans. ALG6‐CDG (*n* = 4), ALG8‐CDG (*n* = 4), DPAGT1‐CDG (*n* = 3), and signal sequence receptor 4 of TRAP complex defect of SSR4‐CDG (*n* = 1) fell in this category. Without having available patient samples, we hypothesize that CDG‐I defects in the oligosaccharyltransferase complex (OSTase), such as defects of TUSC3, DDOST, STT3A, and STT3B will fit in *Group 4*. A summary of the glycomics data of the N‐tetrasaccharide, *N*‐pentasaccharide, and high‐mannose N‐glycans is displayed in Table S1.

### 3.3 Combination of plasma glycomics and high‐throughput genomics to solve CDG‐Ix cases

**FIGURE 3:**
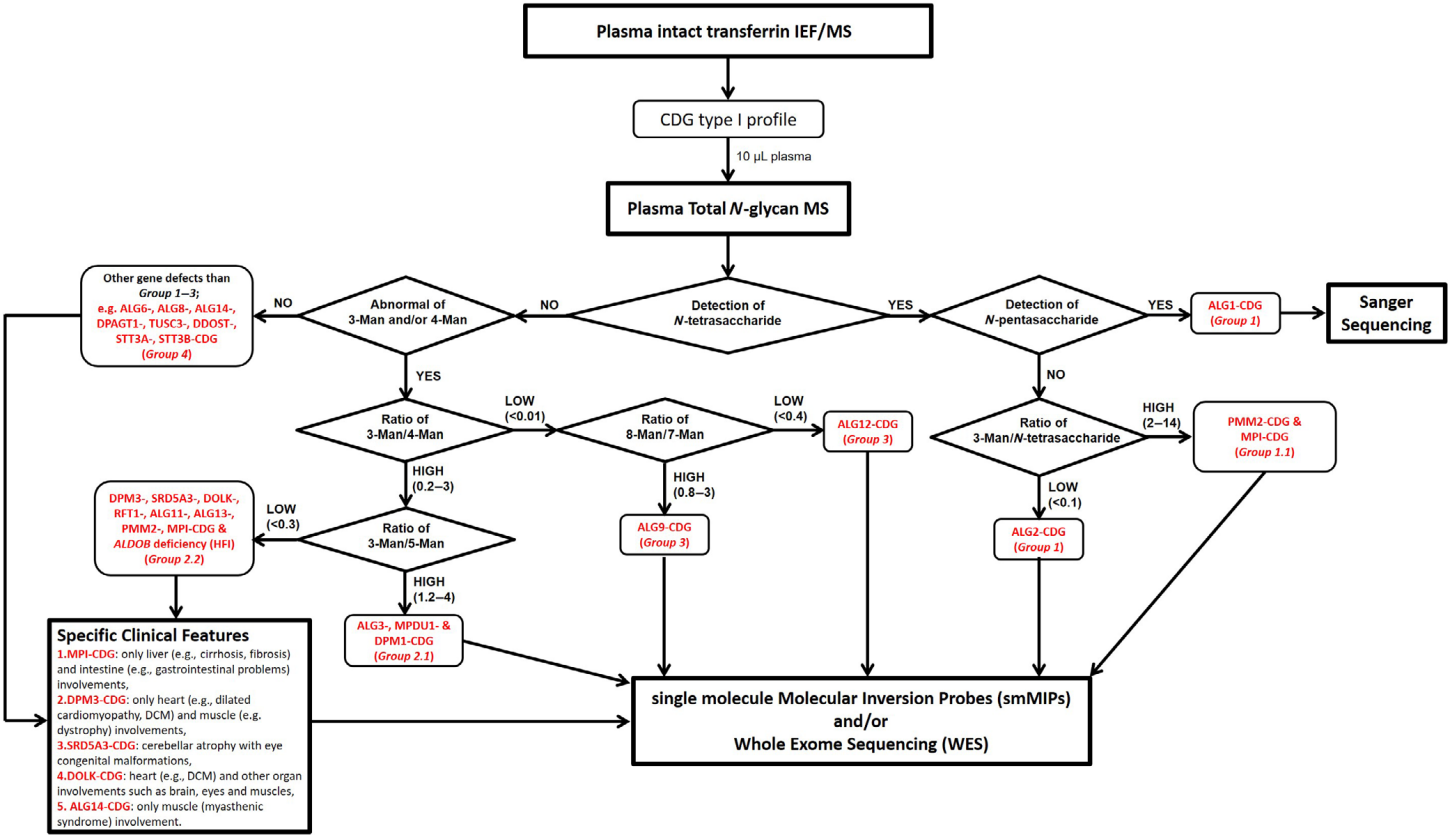
Diagnostic flowchart by combining glycomics, genomics, and clinical signatures to unravel the gene defects in 36 congenital disorders of glycosylation (CDG)‐Ix patients

To validate our glycomics findings, we extended the glycomics analyses to a series of 36 unsolved CDG‐Ix patients (Table S1). Based on the identified glycoprofiles as hallmarks, we designed a diagnostic flowchart (Figure 3) for CDG‐I diagnostics. Since glycomics profiles hinted to a limited number of candidate genes, we developed smMIPs for fast sequencing. In addition, some patients underwent WES, while patients suspected for ALG1‐CDG were confirmed by Sanger sequencing. Depending on available infrastructure, Sanger sequencing might be selected for some individual defects, such as ALG9‐CDG or ALG12‐CDG.

Briefly, we used the following strategy to narrow down the number of candidate genes for patients with unsolved CDG‐I: (a) detection of the N‐tetrasaccharide to define patients in *Group 1*, followed by the detection of *N*‐pentasaccharide for ALG1‐CDG diagnosis and the analysis of the Man3/N‐tetrasaccharide ratio to distinguish ALG2‐CDG from PMM2‐ and MPI‐CDG, and (b) abnormal Man3 and/or Man4 glycans and their ratio to define patients in *Group 2* or in *Group 3*, followed by the analysis of the Man3/Man5 and Man8/Man7 ratios to distinguish Groups 2.1 and 2.2 and for diagnosis of ALG12‐ and ALG9‐CDG, respectively. All patients with normal glycosylation were included in *Group 4*. As depicted in Table 1, glycomics profiling allowed to cluster patients in specific subgroups: (a) four patients suggestive for ALG1‐CDG (*Group 1*), (b) nine patients in *Group 1.1* (PMM2‐ and MPI‐CDG), (c) five patients in *Group 2.1* (ALG3‐, MPDU1‐, and DPM1‐CDG), (d) 12 patients in *Group 2.2* (DPM3‐, SRD5A3‐, DOLK‐, RFT1‐, ALG11‐, ALG13‐, PMM2‐, MPI‐CDG, and HFI), (e) one patient suggestive for ALG12‐CDG (*Group 3*), and (f) five patients in *Group 4*. Sanger sequencing of ALG1 confirmed the diagnosis of ALG1‐CDG in three patients. The lack of identified ALG1 mutations in P79 could be related to the presence of genetic variants outside the exons; however, no patients' cells were available to study ALG1 cDNA. For most of the other patients, smMIPs panels were selected for sequencing of the candidate genes, while for 3CDG‐Ix patients, we used available exome data to search for mutations in the causative genes which revealed a diagnosis that fitted with the designated glycomics groups [DPM1‐CDG (P89), DPM3‐CDG (P94), and ALG12‐CDG (P106)]. smMIPs panel sequencing resulted in the identification of genetic variants in 23 of the 29 sequenced patients. One of the still unsolved patients was present in *Group 1.1*, five were present in *Group 2.2*, while one was in *Group 4*. In summary, combination of glycomics with targeted gene sequencing resolved the diagnosis in 28 of the 36 unsolved CDG‐Ix cases (78%).

**TABLE 1:** Overview of glycomics and genomics data of 36 CDG‐Ix patients

No	Patient's ID	Glycomics classification	Gene	Genomics	cDNA variant	Amino acid change	Genotype
1	P76_CDGIx	ALG1‐CDG (*Group 1*)	*ALG1*	Sanger	NM_019109.5:c.143G > A NM_019109.5:c.826C > T	p.(Arg48His) p.(Arg276Trp)	Heterozygous Heterozygous
2	P77_CDGIx	ALG1‐CDG (*Group 1*)	*ALG1*	Sanger	NM_019109.5:c.293C > T NM_019109.5:c.1150G > A	p.(Pro98Leu) p.(Gly384Arg)	Heterozygous Heterozygous
3	P78_CDGIx	ALG1‐CDG (*Group 1*)	*ALG1*	Sanger	NM_019109.5:c.826C > T NM_019109.5:c.1051G > T	p.(Arg276Trp) p.(Glu351*)	Heterozygous Heterozygous
4	P79_CDGIx	ALG1‐CDG (*Group 1*)	Unsolved	Sanger	n/a	n/a	n/a
5	P80_CDGIx	*Group 1.1*	*PMM2*	smMIPs	NM_000303.3:c.470T > C NM_000303.3:c.722G > C	p.(Phe157Ser) p.(Cys241Ser)	Heterozygous Heterozygous
6	P81_CDGIx	*Group 1.1*	*PMM2*	smMIPs	NM_000303.3:c.97C > T NM_000303.3:c.484C > T	p.(Gln33*) p.(Arg162Trp)	Heterozygous Heterozygous
7	P82_CDGIx	*Group 1.1*	*PMM2*	smMIPs	NM_000303.3:c.669C > A NM_000303.3:c.710C > G	p.(Asp223Glu) p.(Thr237Arg)	Heterozygous Heterozygous
8	P83_CDGIx	*Group 1.1*	*PMM2*	smMIPs	NM_000303.3:c.422G > A NM_000303.3:c.484C > T	p.(Arg141His) p.(Arg162Trp)	Heterozygous Heterozygous
9	P84_CDGIx	*Group 1.1*	*PMM2*	smMIPs	NM_000303.3:c.422G > A NM_000303.3:c.484C > T	p.(Arg141His) p.(Arg162Trp)	Heterozygous Heterozygous
10	P85_CDGIx	*Group 1.1*	*PMM2*	smMIPs	NM_000303.3:c.422G > A NM_000303.3:c.484C > T	p.(Arg141His) p.(Arg162Trp)	Heterozygous Heterozygous
11	P86_CDGIx	*Group 1.1*	*PMM2*	smMIPs	NM_000303.3:c.470T > C NM_000303.3:c.722G > C	p.(Phe157Ser) p.(Cys241Ser)	Heterozygous Heterozygous
12	P87_CDGIx	*Group 1.1*	*PMM2*	smMIPs	NM_000303.3:c.193G > T NM_000303.3:c.422G > A	p.(Asp65Tyr) p.(Arg141His)	Heterozygous Heterozygous
13	P88_CDGIx	*Group 1.1*	Unsolved	smMIPs	n/a	n/a	n/a
14	P89_CDGIx	*Group 2.1*	*DPM1*	WES	NM_003859.3:c.1A > C NM_003859.3:c.274C > G	p.? (start loss) p.(Arg92Gly)	Heterozygous Heterozygous
15	P90_CDGIx	*Group 2.1*	*MPDU1*	smMIPs	NM_004870.4:c.532_534del	p.(His178del)	Homozygous
16	P91_CDGIx	*Group 2.1*	*MPDU1*	smMIPs	NM_004870.4:c.532_534del	p.(His178del)	Homozygous
17	P92_CDGIx	*Group 2.1*	*MPDU1*	smMIPs	NM_004870.4:c.69del	p.(Tyr23*)	Homozygous
18	P93_CDGIx	*Group 2.1*	*MPDU1*	smMIPs	NM_004870.4:c.69del	p.(Tyr23*)	Homozygous
19	P94_CDGIx	*Group 2.2*	*DPM3*	WES	NM_018973.3:c.344T > C	p.(Leu115Ser)	Homozygous
20	P95_CDGIx	*Group 2.2*	*SRD5A3*	smMIPs	NM_024592.5:c.460T > C	p.(Ser154Pro)	Homozygous
21	P96_CDGIx	*Group 2.2*	*SRD5A3*	smMIPs	NM_024592.5:c.697 + 1G > C	effect on splicing	Homozygous
22	P97_CDGIx	*Group 2.2*	*SRD5A3*	smMIPs	NM_024592.5:c.32del NM_024592.5:c.697 + 1G > C	p.(Ala11Glyfs*2) splicing effect	Heterozygous Heterozygous
23	P98_CDGIx	*Group 2.2*	*DOLK*	smMIPs	NM_014908.4:c.3G > C	loss of start codon	Homozygous
24	P99_CDGIx	*Group 2.2*	*PMM2*	smMIPs	NM_000303.3:c.422G > A NM_000303.3:c.722G > C	p.(Arg141His) p.(Cys241Ser)	Heterozygous Heterozygous
25	P100_CDGIx	*Group 2.2*	*ALG11*	smMIPs	NM_001004127.3:c.254C > T NM_001004127.3:c.1294G > T	p.(Ala85Val) p.(Gly432*)	Heterozygous Heterozygous
26	P101_CDGIx	*Group 2.2*	Unsolved	smMIPs	n/a	n/a	n/a
27	P102_CDGIx	*Group 2.2*	Unsolved	smMIPs	n/a	n/a	n/a
28	P103_CDGIx	*Group 2.2*	Unsolved	smMIPs	n/a	n/a	n/a
29	P104_CDGIx	*Group 2.2*	Unsolved	smMIPs	n/a	n/a	n/a
30	P105_CDGIx	*Group 2.2*	Unsolved	smMIPs	n/a	n/a	n/a
31	P106_CDGIx	ALG12‐CDG (*Group 3*)	*ALG12*	WES	NM_024105.4:c.233C > T NM_024105.4:c.295 + 1G > A	p.(Ser78Phe) effect on splicing	Heterozygous Heterozygous
32	P107_CDGIx	*Group 4*	*DPAGT1*	smMIPs	NM_001382.4:c.988G > A	p.(Gly330Ser)	Homozygous
33	P108_CDGIx	*Group 4*	*DPAGT1*	smMIPs	NM_001382.4:c.988G > A	p.(Gly330Ser)	Homozygous
34	P109_CDGIx	*Group 4*	*ALG6*	smMIPs	NM_013339.4:c.257 + 5G > A NM_013339.4:c.998C > T	effect on splicing p.(Ala333Val)	Heterozygous Heterozygous
35	P110_CDGIx	*Group 4*	Unsolved	smMIPs	n/a	n/a	n/a
36	P111_CDGIx	*Group 4*	*ALG6*	smMIPs	NM_013339.4:c.391T > C	p.(Tyr131His)	Heterozygous

Abbreviations: CDG, congenital disorders of glycosylation; cDNA, complementary DNA; ID, identification; n/a: not available; P, patient; smMIP, single‐molecule molecular inversion probes; WES, whole exome sequencing.

### 3.4 Summary of clinical data in the solved cases (28 of 36) of CDG‐Ix study

The clinical features of the solved cases showed symptoms that are in general agreement with CDG‐I. The majority of cases showed an overall clinical course of neurological symptoms with multisystem involvement. A summary of clinical details of the 28 patients that are solved in this study is provided in Table S2.

One of the most frequent neurological symptoms is psychomotor delay/mental deficiency (PD/MD), which is present in all diseases reported in the present study, except in DPM3‐CDG. Severe MD was present in most diseases, while mild or moderate PD/MD was found only in ALG12‐, PMM2‐, and DPAGT1‐CDG individuals. Brain atrophy leading to microcephaly was present in most diseases with MD. Only DPAGT1‐ and SRD5A3‐CDG patients showed normal cephalic perimeters. Cerebellar atrophy and ataxia were found only in ALG1‐CDG and PMM2‐CDG, being present in almost all patients in the latter. In DPAGT1‐CDG, ataxia was not accompanied by cerebellar atrophy.

Except for DPM3‐CDG, hypotonia was one of the most frequent findings, present in most patients in all other diseases, occasionally accompanied by pyramidal symptoms or dystonia. Seizures were present in most CDG patients, except in those with ALG12‐, DPM3‐, SRD5A3‐, and DPAGT‐CDG.

Muscle weakness was present in ALG1‐, ALG11‐, and DPGAT1‐CDG. In ALG12‐CDG, muscle weakness was associated with dystonia, aortic myopathy with dilation, tracheomalacia, and bronchiectasis, composing a very distinctive clinical picture. The presentation of DPM3‐CDG is that of a “pure” myopathy (including dilated cardiomyopathy), with a marked increase of serum CK. Hypertrophic myocardiopathy was seen in DOLK‐CDG without signs of systemic myopathy.

Hepatomegaly was present in ALG1‐, ALG6‐, DOLK‐, and SRD5A3‐CDG. Persistent diarrhea seems to be a striking feature in ALG1‐CDG.

Facial dysmorphisms were found in all diseases of this study, except in ALG12‐, DPM3‐, and DPAGT1‐CDG. Dysphagia and/or gastroesophageal reflux was present in ALG1‐, ALG6‐, ALG11‐, ALG12‐, DPM1‐, and DOLK‐CDG.

Some findings were distinctive of some particular diseases. Limb shortening was present only in ALG1‐CDG, while involuntary limb movements and oral dyskinesis seem to be the characteristic of DOLK‐CDG. Melanocytic lesions (i.e., acantosis nigricans) were found in ALG1 and SRD5A3 patients, associated with dermatitis in the latter. Eyes abnormalities such as glaucoma and buphthalmia were present in SRD5A3 patients, and optic nerve/disc alterations are a relatively nonspecific finding, present in ALG6‐, DPM1‐, MPDU‐, and SRD5A3‐CDG.

## 4 DISCUSSION

A high degree of clinical heterogeneity and a lack of specific biochemical markers are current challenges for diagnosing patients with CDG‐I. In this study, we have identified novel and specific CDG‐I glycomarkers in a majority of CDG‐I types including HFI (16 gene defects). Subsequent establishment of targeted sequencing using smMIPs resulted in correct diagnosis in 78% of the CDG‐I patients.

**FIGURE 4:**
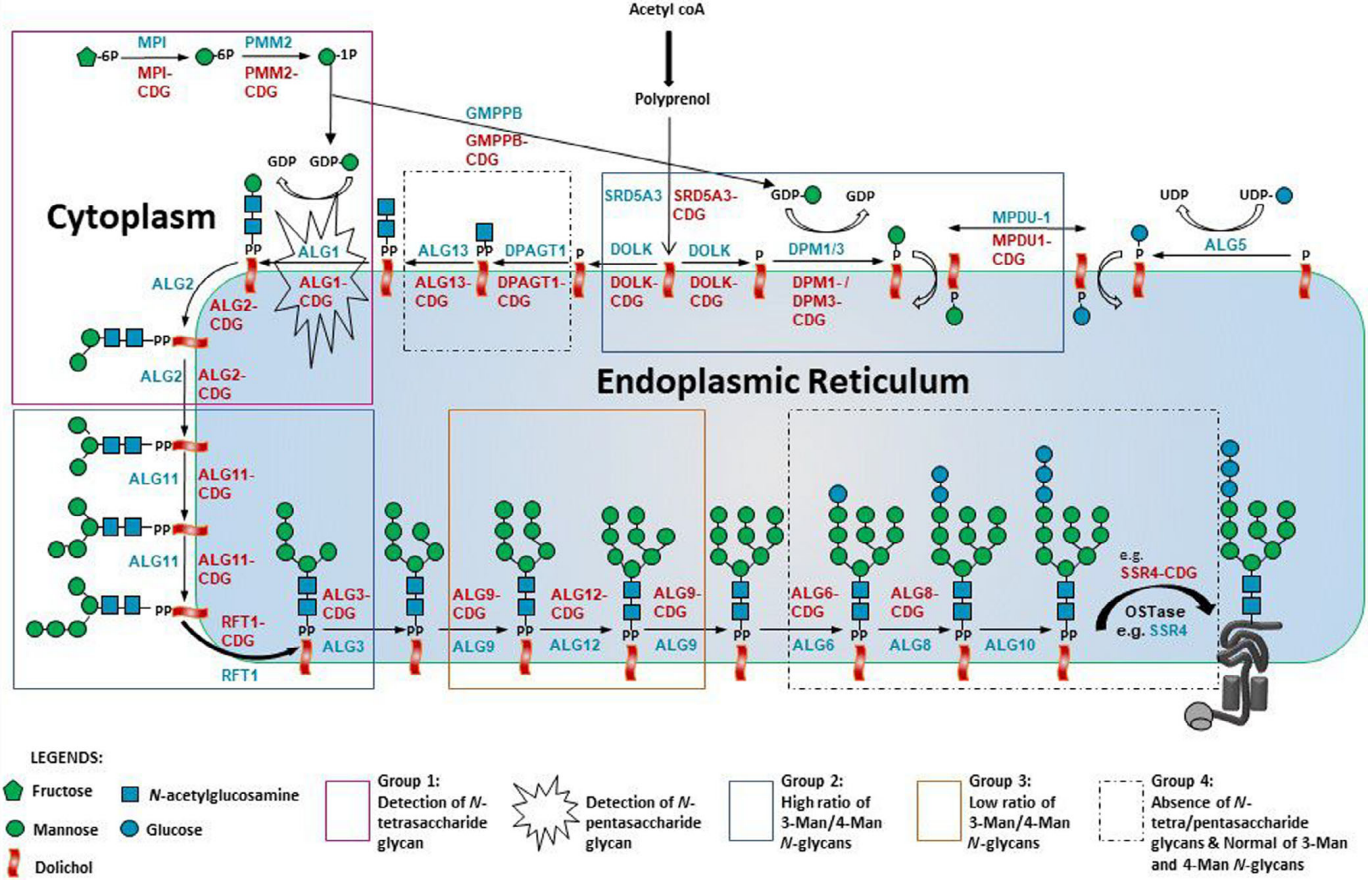
N‐glycosylation pathway with indicated glycan abnormalities in congenital disorders of glycosylation type 1 (CDG‐I) defects. Four groups of gene defects (Groups 1–4) were determined on the basis of their total plasma glycoprofiling. A blue font color represents affected genes while a red font color represents a type of CDG on basis of standard CDG nomenclature

The origin of the abnormal glycan structures in CDG‐I gene defects derived from this study (Figure 4) can partially be understood. In a previous study, the presence of an N‐tetrasaccharide in plasma of ALG1‐CDG patients was explained by the accumulation of dolichol‐linked chitobiose (GlcNAc_2_) in the ER as a result of deficient mannosylation by ALG1.^7^ This abnormal glycan precursor is subsequently transferred to nascent proteins (*N*‐linked chitobiose) and further modified in the Golgi apparatus to form the galactosylated and sialylated N‐tetrasaccharide (Neu5Ac_1_Gal_1_GlcNAc_2_). In the current study, a novel fucosylated pentasaccharide (Neu5Ac_1_Gal_1_(Fuc)GlcNAc_2_) was identified in ALG1‐CDG. Confirmation by MS/MS fragmentation that the fucose was linked to the nonterminal GlcNAc residue is a confirmation of the hypothesized biosynthesis pathway, in which the chitobiose core is further modified by Golgi glycosyltransferases, including a fucosyltransferase. The pentasaccharide was not identified in the other defects of *Group 1* patients, even if ALG2‐CDG presented with similar increases of the N‐tetrasaccharide. For ALG12‐CDG, accumulation of Man7 glycans was specific for the diagnosis. The ALG12 mannosyltransferase is required to convert Man_7_GlcNAc_2_ to Man_8_GlcNAc_2_. Its deficiency will result in accumulation of the Man_7_GlcNAc_2_‐PP‐dolichol precursor and its subsequent transfer to nascent glycoproteins. Apparently, the Man7 glycan is not further processed in the Golgi, which results in secreted glycoproteins with Man7 high‐mannose glycans in patient plasma.

In previous studies, accumulation of dolichol‐linked Man_3_GlcNAc_2_ and Man_4_GlcNAc_2_ glycans was hypothesized due to reduced GDP‐mannose availability in PMM2‐CDG and MPI‐CDG.^7^, ^9^ Subsequent transfer to proteins would explain the increased detection of Man3 and Man4 glycans in patient plasma. As depicted in Figure 4, our study indicated quite a number of additional CDG‐I defects with accumulation of Man3 and/or Man4 glycans including mannosyltransferase deficiencies (ALG11‐, ALG3‐, ALG9‐, and ALG12‐CDG), flippase deficiency (RFT1‐CDG), and defects in the synthesis and utilization of dolichol‐phosphate mannose (SRD5A3‐, DOLK‐, DPM3‐, DPM1‐, and MPDU1‐CDG). Interestingly, all of these defects are related to the incorporation of mannose residues into the DLO. Defects in dolichol‐phosphate mannose synthesis and in ALG3 would result in the accumulation of Man5 glycans. In line with the hypothesis on the biosynthetic pathway, such glycans will be transferred to proteins and an accumulation of Man5 glycans is expected. However, mainly Man3 glycans accumulate in these CDG‐I defects. The data could thus indicate that translated Man5 glycans are processed by ER and/or Golgi‐resident mannosidases to Man3 glycans. It is interesting to note that ALG9 and ALG12 defects do not show accumulation of Man3 glycans but instead show elevated levels of Man4 glycans. This could indicate that the different high‐mannose isomers are differently processed by ER and Golgi mannosidases; however, this requires further investigation. Defects other than “mannose‐related defects,” such as glucosyltransferase defects (ALG6‐ and ALG8‐CDG), GlcNAc transferase defects (DPAGT1‐ and ALG13‐CDG), and defects in the oligosaccharyltransferase complex (SSR4‐CDG) exhibited normal levels of Man3 and Man4 glycans (*Group 4*).

For many years, transferrin has been a useful marker for diagnosis of CDG‐I. Using MS, it allows highly sensitive detection of a reduction in site‐occupancy, which is characteristic for CDG‐I defects. Minor traces of the N‐tetrasaccharide can be detected on the transferrin protein only in ALG1‐CDG by MS.^7^, ^17^ However, the other glycan structural abnormalities such as the *N*‐pentasaccharide and the accumulation of Man3 and Man4 glycans in PMM2 and MPI‐CDG are undetectable on transferrin. Hence, this protein marker is not ideal to identify the more subtle glycan abnormalities as found via glycomics. Application of glycomics could be used as a second‐tier test after transferrin screening to obtain this information. Thus far, the majority of studies on glycomics in CDG have exploited profiling methodologies, such as MALDI‐TOF and LC‐QTOF MS. For ALG3‐CDG, increased abundance of short high‐mannose glycans were found for four patients including Man3 and Man4 structures.^18^ Application in single cases revealed a lowered ratio of Man8/Man7 glycans for ALG12‐CDG, similar to the current study.^19^ Standardization of methodology for absolute quantification might further aid positioning of glycomics as diagnostic test.^9^ Further validation of the potential biomarkers reported before and in the current study is necessary by the establishment of well‐defined patient cohorts with standardized collection of biomaterials, consisting of sufficient number of patient samples per defect and in time.

Alternatively, a selection of protein markers could be added for CDG diagnostics to profile glycosylation signatures such as N‐glycosylation profiles of several major plasma glycoproteins of alpha‐1‐antitrypsin, alpha‐2‐macroglobulin, apolipoproteins, and immunoglobulins.^20^, ^21^ However, it is not yet known what other proteins will contain the identified glycan abnormalities and why other glycoproteins are more sensitive to display such a defect. Possibly, the efficiency of the transfer of intermediate glycan structures to a protein is dependent on protein sequence and/or structure. Therefore, further glycoproteomics studies are required to identify novel potential protein markers to increase the diagnostic specificity for CDG‐I gene identification.

In conclusion, we observed abnormal glycan profiles in 16 genetic defects associated with CDG‐I (including HFI), which facilitates diagnosis and may aid therapy monitoring in future clinical trials. Abnormal glycan profiles can be followed by sequencing of the respective candidate genes or inspecting exome sequencing results or can be used to confirm already identified mutations. In combination with specific clinical symptoms and by targeted smMIPs sequencing, plasma glycomics profiling facilitates identification and confirmation of CDG‐I gene defects.

## CONFLICT OF INTEREST

All the authors declare that they have no conflict of interest and no financial relationships relevant to this article to disclose

## AUTHOR CONTRIBUTIONS

Dirk J. Lefeber, Nurulamin Abu Bakar, Angel Ashikov, Jaime Moritz Brum, Lambertus P. van den Heuvel, and Christian Thiel designed the study. Nurulamin Abu Bakar, Angel Ashikov, Jaime Moritz Brum, Roel Smeets, Marjan Kersten, Karin Huijben, Wee Teik Keng, Carlos Speck‐Martins, Daniel Rocha de Carvalho, Isabela Maria Pinto de Oliveira Rizzo, Walquiria Domingues de Mello, M. Rebecca Heiner‐Fokkema, Kathleen Gorman, Helen Michelakakis, Marina Moraitou, and Diego Martinelli conducted the study and acquired data. Dirk J. Lefeber, Nurulamin Abu Bakar, Angel Ashikov, Jaime Moritz Brum, Roel Smeets, Marjan Kersten, Wee Teik Keng, M. Rebecca Heiner‐Fokkema, Stephanie Grunewald, Monique van Scherpenzeel, Mirian Janssen, Lonneke de Boer, Lambertus P. van den Heuvel, and Christian Thiel interpreted the data. Dirk J. Lefeber and Nurulamin Abu Bakar wrote the first draft of the manuscript. Finally, all authors edited and reviewed the manuscript.

## ETHICS STATEMENT

All procedures followed were in accordance with the ethical standards of the responsible committee on human experimentation (institutional and national) and with the Helsinki Declaration of 1975, as revised in 2000. Diagnostic procedures for informed consent were followed for sequencing of genes and CDG screening in plasma, informed consent was obtained from all patients for clinical data according to the regulations of the participating institutions and use of diagnostic plasma samples for glycomics profiling was approved by the local institutional review board (2019‐5591).

## Supporting information


**Supplementary Table 1** Glycomics data of 111 CDG‐I patients with known (*n* = 75) and unsolved (36) genetic cause. The relative abundances are shown of the N‐tetrasaccharide, the N‐pentasaccharide and a series of high mannose glycans.

**Supplementary Table 2** Clinical data of the 28 CDG‐I patients that were genetically solved in this study.

## Data Availability

The data that supports the findings of this study are available in the Supporting Information of this article
