# Supplementary material for: Synergistic use of glycomics and single‐molecule molecular inversion probes for identification of congenital disorders of glycosylation type‐1
Source: J Inherit Metab Dis. 2022 Mar 28;45(4):769–81. doi: 10.1002/jimd.12496 (PMC9545396; doi:10.1002/jimd.12496)
Supplement: Supplementary file 1 — Supplementary Table 1 Glycomics data of 111 CDG‐I patients with known (n = 75) and unsolved (36) genetic cause. The relative abundances are shown of the N‐tetrasaccharide, the N‐pentasaccharide and a series of high mannose glycans. [file JIMD-45-769-s002.docx]

| **confidence intervals (n=40)** | **(not detected)** | **(not detected)** | **(less than 0.04)** | **(0.04-0.09)** | **(2.19-2.68)** | **(1.76-2.11)** | **(0.48-0.56)** | **(0.62-0.73)** | **(0.58-0.69)** |
| --- | --- | --- | --- | --- | --- | --- | --- | --- | --- |
| **Patient ID_Gene defects / ^#^unsolved** | **4-saccharide** | **5-saccharide** | **Man_3_GlcNAc_2_** | **Man_4_GlcNAc_2_** | **Man_5_GlcNAc_2_** | **Man_6_GlcNAc_2_** | **Man_7_GlcNAc_2_** | **Man_8_GlcNAc_2_** | **Man_9_GlcNAc_2_** |
| P1_PMM2 | 0.66 | 0 | 1.38 | 2.93 | 2.28 | 0.74 | 0.03 | 0.04 | 0.03 |
| P2_PMM2 | 0.14 | 0 | 1.23 | 1.26 | 1.41 | 0.67 | 0.17 | 0.16 | 0.06 |
| P3_PMM2 | 0.6 | 0 | 2.96 | 3.69 | 2.7 | 0.86 | 0.09 | 0.07 | 0.13 |
| P4_PMM2 | 0.54 | 0 | 2.25 | 2.4 | 2.02 | 0.5 | 0.04 | 0.03 | 0.02 |
| P5_PMM2 | 0.22 | 0 | 1.18 | 0.96 | 1.74 | 0.76 | 0.07 | 0.1 | 0.19 |
| P6_PMM2 | 0.25 | 0 | 0.71 | 0.81 | 2.2 | 1.1 | 0.26 | 0.28 | 0.15 |
| P7_PMM2 | 0.52 | 0.02 | 2.03 | 1.86 | 1.35 | 0.52 | 0.03 | 0 | 0 |
| P8_PMM2 | 0.22 | 0 | 0.89 | 1.13 | 1.03 | 0.27 | 0 | 0 | 0 |
| P9_PMM2 | 0.26 | 0 | 1.92 | 1.93 | 2.1 | 1.22 | 0.26 | 0.21 | 0.22 |
| P10_PMM2 | 0.2 | 0 | 1.04 | 1.58 | 2.18 | 1.2 | 0.16 | 0.22 | 0.13 |
| P11_PMM2 | 0 | 0 | 0.31 | 0.27 | 1.51 | 0.93 | 0.16 | 0.25 | 0.31 |
| P12_MPI | 0.12 | 0 | 1.22 | 1.23 | 2.77 | 1.33 | 0.32 | 0.26 | 0.35 |
| P13_MPI | 0.16 | 0 | 0.44 | 0.66 | 1.3 | 0.22 | 0 | 0 | 0.02 |
| P14_MPI | 0.08 | 0 | 0.35 | 0.3 | 1.65 | 0.44 | 0.01 | 0.02 | 0.13 |
| P15_MPI | 0.03 | 0 | 0.38 | 0.58 | 1.42 | 0.6 | 0.01 | 0.03 | 0.11 |
| P16_MPI | 0 | 0 | 0.11 | 0.25 | 0.99 | 0.5 | 0.01 | 0 | 0.06 |
| P17_ALG1 | 5.28 | 0.25 | 0.04 | 0.03 | 1.66 | 0.86 | 0.12 | 0.12 | 0.14 |
| P18_ALG1 | 5.29 | 0.43 | 0.1 | 0.13 | 1.45 | 0.84 | 0.09 | 0.06 | 0.12 |
| P19_ALG1 | 1.83 | 0.09 | 0.03 | 0.06 | 1.7 | 1.78 | 0.41 | 0.48 | 0.33 |
| P20_ALG2 | 0.77 | 0 | 0.12 | 0.18 | 1.8 | 0.68 | 0.08 | 0 | 0.09 |
| P21_ALG2 | 1.4 | 0 | 0 | 0 | 1.78 | 0.99 | 0.18 | 0.22 | 0.18 |
| P22_ALG3 | 0 | 0 | 2.93 | 1.28 | 0.94 | 0.59 | 0.04 | 0.04 | 0.02 |
| P23_ALG3 | 0 | 0 | 2.64 | 1.21 | 0.76 | 0.22 | 0 | 0 | 0 |
| P24_ALG3 | 0 | 0 | 6.2 | 3.03 | 1.57 | 0.63 | 0.04 | 0.02 | 0 |
| P25_ALG3 | 0 | 0 | 2.96 | 1.51 | 1.6 | 0.55 | 0.04 | 0.09 | 0.16 |
| P26_ALG3 | 0 | 0 | 3.33 | 1.61 | 1.44 | 0.66 | 0.01 | 0.06 | 0.05 |
| P27_MPDU1 | 0 | 0 | 5.41 | 3.09 | 2.09 | 0.47 | 0 | 0 | 0 |
| P28_DPM1 | 0 | 0 | 2.93 | 2.71 | 1.96 | 0.84 | 0.12 | 0.05 | 0.01 |
| P29_DPM3 | 0 | 0 | 1.72 | 1.5 | 5.42 | 2.92 | 0.54 | 0.51 | 0.36 |
| P30_SRD5A3 | 0 | 0 | 0.85 | 0.78 | 3.52 | 1.44 | 0.2 | 0.23 | 0.37 |
| P31_SRD5A3 | 0 | 0 | 0.51 | 0.51 | 1.82 | 0.76 | 0.07 | 0.09 | 0.2 |
| P32_SRD5A3 | 0 | 0 | 1.08 | 1.32 | 4.3 | 1.55 | 0.31 | 0.18 | 0.3 |
| P33_SRD5A3 | 0 | 0 | 1.01 | 0.9 | 3.14 | 1.51 | 0.24 | 0.26 | 0.3 |
| P34_SRD5A3 | 0 | 0 | 0.53 | 0.43 | 3.09 | 1.56 | 0.18 | 0.35 | 0.4 |
| P35_SRD5A3 | 0 | 0 | 0.13 | 0.07 | 2.29 | 0.82 | 0.11 | 0.18 | 0.23 |
| P36_SRD5A3 | 0 | 0 | 0.63 | 0.7 | 3.18 | 1.75 | 0.34 | 0.36 | 0.42 |
| P37_SRD5A3 | 0 | 0 | 0.54 | 0.8 | 2.27 | 1.17 | 0.15 | 0.21 | 0.28 |
| P38_SRD5A3 | 0 | 0 | 0.04 | 0.06 | 2.87 | 1.51 | 0.34 | 0.52 | 0.51 |
| P39_SRD5A3 | 0 | 0 | 0.06 | 0.08 | 2.32 | 1.23 | 0.26 | 0.44 | 0.52 |
| P40_DOLK | 0 | 0 | 0.11 | 0.13 | 1.7 | 1.19 | 0.06 | 0 | 0.32 |
| P41_DOLK | 0 | 0 | 0.1 | 0.14 | 1.5 | 1.05 | 0.13 | 0.18 | 0.21 |
| P42_DOLK | 0 | 0 | 0.26 | 0.45 | 3.3 | 2.64 | 0.43 | 0.44 | 0.4 |
| P43_DOLK | 0 | 0 | 0.11 | 0.15 | 1.88 | 1.19 | 0.22 | 0.28 | 0.32 |
| P44_DOLK | 0 | 0 | 0.13 | 0.2 | 3.77 | 2.83 | 0.45 | 0.49 | 0.49 |
| P45_DOLK | 0 | 0 | 0.39 | 0.19 | 3.68 | 1.1 | 0.06 | 0.12 | 0.16 |
| P46_DOLK | 0 | 0 | 0.06 | 0.19 | 1.69 | 0.92 | 0.09 | 0.18 | 0.27 |
| P47_DOLK | 0 | 0 | 0.3 | 0.32 | 3.06 | 1.5 | 0.22 | 0.35 | 0.47 |
| P48_RFT1 | 0 | 0 | 0.14 | 0.25 | 4.07 | 2.51 | 0.5 | 0.49 | 0.77 |
| P49_RFT1 | 0 | 0 | 0.06 | 0.12 | 3.46 | 2.21 | 0.45 | 0.45 | 0.41 |
| P50_ALG11 | 0 | 0 | 0.13 | 0.3 | 3.89 | 1.12 | 0.14 | 0.23 | 0.33 |
| P51_ALG11 | 0 | 0 | 0.12 | 0.18 | 2.58 | 0.76 | 0.1 | 0.1 | 0.21 |
| P52_ALDOB | 0 | 0 | 0.12 | 0.11 | 3.74 | 1.82 | 0.43 | 0.44 | 0.36 |
| P53_ALDOB | 0 | 0 | 0.34 | 0.36 | 2.55 | 1.09 | 0.27 | 0.33 | 0.43 |
| P54_ALDOB | 0 | 0 | 0.1 | 0.17 | 2.82 | 1.71 | 0.39 | 0.41 | 0.5 |
| P55_ALDOB | 0 | 0 | 0.24 | 0.37 | 2.84 | 2.15 | 0.32 | 0.4 | 0.55 |
| P56_ALG9 | 0 | 0 | 0 | 1.27 | 2.63 | 1.3 | 0.33 | 0.39 | 0.18 |
| P57_ALG9 | 0 | 0 | 0.03 | 2.77 | 3.56 | 1.45 | 0.18 | 0.15 | 0.01 |
| P58_ALG9 | 0 | 0 | 0 | 2.38 | 3.43 | 1.32 | 0.03 | 0.09 | 0.03 |
| P59_ALG12 | 0 | 0 | 0 | 0.83 | 3.01 | 1.99 | 0.72 | 0.05 | 0 |
| P60_ALG12 | 0 | 0 | 0 | 0.92 | 3.55 | 1.7 | 0.56 | 0.07 | 0.03 |
| P61_ALG12 | 0 | 0 | 0 | 0.73 | 3.24 | 1.49 | 0.25 | 0.09 | 0.16 |
| P62_ALG12 | 0 | 0 | 0 | 1.52 | 4.57 | 1.32 | 0.1 | 0 | 0 |
| P63_DPAGT1 | 0 | 0 | 0 | 0.02 | 2.74 | 1.53 | 0.07 | 0.17 | 0.14 |
| P64_DPAGT1 | 0 | 0 | 0 | 0 | 1.87 | 1 | 0.08 | 0.16 | 0.21 |
| P65_DPAGT1 | 0 | 0 | 0 | 0 | 1.97 | 1.25 | 0.17 | 0.36 | 0.48 |
| P66_ALG6 | 0 | 0 | 0 | 0.08 | 3.78 | 1.63 | 0.17 | 0.31 | 0.37 |
| P67_ALG6 | 0 | 0 | 0 | 0.03 | 2.45 | 1.42 | 0.09 | 0.25 | 0.39 |
| P68_ALG6 | 0 | 0 | 0 | 0.03 | 2.06 | 0.93 | 0.08 | 0.18 | 0.25 |
| P69_ALG6 | 0 | 0 | 0 | 0.07 | 2.28 | 0.81 | 0.04 | 0.09 | 0.17 |
| P70_ALG8 | 0 | 0 | 0 | 0 | 1.38 | 0.76 | 0.07 | 0.17 | 0.34 |
| P71_ALG8 | 0 | 0 | 0 | 0 | 1.31 | 0.64 | 0.07 | 0.21 | 0.4 |
| P72_ALG8 | 0 | 0 | 0 | 0.08 | 2.89 | 1.87 | 0.18 | 0.37 | 0.5 |
| P73_ALG8 | 0 | 0 | 0 | 0 | 1.9 | 1.27 | 0.14 | 0.26 | 0.38 |
| P74_ALG13 | 0 | 0 | 0.02 | 0.1 | 4.06 | 2.42 | 0.32 | 0.63 | 0.59 |
| P75_SSR4 | 0 | 0 | 0 | 0 | 2.05 | 1.23 | 0.06 | 0.09 | 0.07 |
| ^#^P76_CDGIx | 4.63 | 0.31 | 0.07 | 0.06 | 1.62 | 0.71 | 0.08 | 0.1 | 0.08 |
| ^#^P77_CDGIx | 2.42 | 0.13 | 0.07 | 0.13 | 2.51 | 1.59 | 0.37 | 0.51 | 0.35 |
| ^#^P78_CDGIx | 4.69 | 0.39 | 0.06 | 0.08 | 2.12 | 1.08 | 0.21 | 0.25 | 0.2 |
| ^#^P79_CDGIx | 0.54 | 0.04 | 1.75 | 0.56 | 1.44 | 0.48 | 0.07 | 0.09 | 0.06 |
| ^#^P80_CDGIx | 0.12 | 0 | 1.26 | 0.5 | 1.98 | 0.94 | 0.2 | 0.29 | 0.17 |
| ^#^P81_CDGIx | 0.15 | 0 | 2.22 | 0.89 | 2.62 | 1.11 | 0.2 | 0.22 | 0.16 |
| ^#^P82_CDGIx | 0.07 | 0 | 1.12 | 0.7 | 1.87 | 0.77 | 0.2 | 0.27 | 0.16 |
| ^#^P83_CDGIx | 0.2 | 0 | 1.44 | 0.61 | 1.98 | 0.82 | 0.17 | 0.18 | 0.08 |
| ^#^P84_CDGIx | 0.06 | 0 | 0.8 | 0.41 | 1.88 | 1.12 | 0.28 | 0.35 | 0.27 |
| ^#^P85_CDGIx | 0.04 | 0 | 0.67 | 0.42 | 2.16 | 0.99 | 0.34 | 0.41 | 0.33 |
| ^#^P86_CDGIx | 0.06 | 0 | 0.47 | 0.23 | 2.01 | 0.69 | 0.2 | 0.26 | 0.17 |
| ^#^P87_CDGIx | 0.15 | 0 | 1.29 | 0.93 | 2.1 | 0.88 | 0.24 | 0.21 | 0.15 |
| ^#^P88_CDGIx | 0.1 | 0 | 0.26 | 0.32 | 2.04 | 1.03 | 0.16 | 0.31 | 0.53 |
| ^#^P89_CDGIx | 0 | 0 | 1.69 | 1.25 | 1.45 | 0.48 | 0.02 | 0.06 | 0.08 |
| ^#^P90_CDGIx | 0 | 0 | 1.05 | 0.88 | 0.49 | 0.2 | 0 | 0 | 0 |
| ^#^P91_CDGIx | 0 | 0 | 4.93 | 2.11 | 2.27 | 0.94 | 0.08 | 0.04 | 0.06 |
| ^#^P92_CDGIx | 0 | 0 | 1.9 | 1.07 | 1.1 | 0.46 | 0.07 | 0.07 | 0.08 |
| ^#^P93_CDGIx | 0 | 0 | 1.7 | 0.97 | 1.15 | 0.48 | 0 | 0.03 | 0 |
| ^#^P94_CDGIx | 0 | 0 | 0.67 | 0.79 | 2.07 | 1.45 | 0.29 | 0.28 | 0.08 |
| ^#^P95_CDGIx | 0 | 0 | 0.7 | 0.72 | 2.22 | 1.34 | 0.26 | 0.31 | 0.34 |
| ^#^P96_CDGIx | 0 | 0 | 0.58 | 0.39 | 3.19 | 1.58 | 0.45 | 0.53 | 0.3 |
| ^#^P97_CDGIx | 0 | 0 | 0.4 | 0.26 | 2.14 | 0.98 | 0.17 | 0.2 | 0.18 |
| ^#^P98_CDGIx | 0 | 0 | 0.43 | 0.26 | 2.3 | 1.3 | 0.28 | 0.37 | 0.02 |
| ^#^P99_CDGIx | 0 | 0 | 0 | 0 | 1.45 | 0.85 | 0 | 0.04 | 0.18 |
| ^#^P100_CDGIx | 0 | 0 | 0.04 | 0.08 | 4.52 | 2.28 | 0.76 | 0.79 | 0.71 |
| ^#^P101_CDGIx | 0 | 0 | 0.05 | 0.13 | 5.22 | 2.13 | 0.83 | 0.62 | 0.76 |
| ^#^P102_CDGIx | 0 | 0 | 0.44 | 0.58 | 2.72 | 1.56 | 0.43 | 0.44 | 0.44 |
| ^#^P103_CDGIx | 0 | 0 | 1.42 | 2.38 | 2.02 | 0.41 | 0.03 | 0 | 0.04 |
| ^#^P104_CDGIx | 0 | 0 | 0.02 | 0.11 | 3.08 | 1.78 | 0.18 | 0.32 | 0.27 |
| ^#^P105_CDGIx | 0 | 0 | 0.11 | 0.15 | 3.18 | 1.71 | 0.34 | 0.47 | 0.51 |
| ^#^P106_CDGIx | 0 | 0 | 0 | 1.54 | 4.45 | 2.08 | 0.65 | 0.15 | 0 |
| ^#^P107_CDGIx | 0 | 0 | 0 | 0.01 | 2.16 | 0.76 | 0.21 | 0.24 | 0.01 |
| ^#^P108_CDGIx | 0 | 0 | 0 | 0.03 | 2.69 | 1.23 | 0.38 | 0.5 | 0.37 |
| ^#^P109_CDGIx | 0 | 0 | 0 | 0 | 0.73 | 0.24 | 0 | 0 | 0.26 |
| ^#^P110_CDGIx | 0 | 0 | 0 | 0 | 2.47 | 1.66 | 0.47 | 0.76 | 0.84 |
| ^#^P111_CDGIx | 0 | 0 | 0.01 | 0.01 | 2.3 | 1.27 | 0.26 | 0.31 | 0.38 |
